# A Non-Motor Microtubule Binding Site Is Essential for the High Processivity and Mitotic Function of Kinesin-8 Kif18A

**DOI:** 10.1371/journal.pone.0027471

**Published:** 2011-11-10

**Authors:** Monika I. Mayr, Marko Storch, Jonathon Howard, Thomas U. Mayer

**Affiliations:** 1 Department of Biology and Konstanz Research School Chemical Biology, University of Konstanz, Konstanz, Germany; 2 Max Planck Institute of Molecular Cell Biology and Genetics, Dresden, Germany; University of Virginia, United States of America

## Abstract

**Background:**

Members of the kinesin-8 subfamily are plus end-directed molecular motors that accumulate at the plus-ends of kinetochore-microtubules (kt-MTs) where they regulate MT dynamics. Loss of vertebrate kinesin-8 function induces hyperstable MTs and elongated mitotic spindles accompanied by severe chromosome congression defects. It has been reported that the motility of human kinesin-8, Kif18A, is required for its accumulation at the plus tips of kt-MTs.

**Methodology/Findings:**

Here, we investigate how Kif18A localizes to the plus-ends of kt-MTs. We find that Kif18A lacking its C-terminus does not accumulate on the tips of kt-MTs and fails to fulfill its mitotic function. *In vitro* studies reveal that Kif18A possesses a non-motor MT binding site located within its C-proximal 121 residues. Using single molecule measurements we find that Kif18A is a highly processive motor and, furthermore, that the C-terminal tail is essential for the high processivity of Kif18A.

**Conclusion/Significance:**

These results show that Kif18A like its yeast orthologue is a highly processive motor. The ability of Kif18A to walk on MTs for a long distance without dissociating depends on a non-motor MT binding site located at the C-terminus of Kif18A. This C-proximal tail of Kif18A is essential for its plus-end accumulation and mitotic function. These findings advance our understanding of how Kif18A accumulates at the tips of kt-MTs to fulfill its function in mitosis.

## Introduction

The integrity of each organism is intrinsically tied to the faithful distribution of its replicated chromosomes during mitosis. This challenging task is mediated by the mitotic spindle; a cellular machine composed of microtubules (MTs) and associated proteins [1]. Microtubules are dynamic polymers assembled from tubulin heterodimers consisting of alpha and beta tubulin. The head to tail assembly of tubulin dimers leads to the formation of polar MT protofilaments with the alpha and beta subunits exposed at the minus- and plus-ends, respectively. Thirteen protofilaments associate laterally to assemble the MT filament, a hollow cylinder of diameter about 25 nm. MTs display dynamic instability, i.e. they undergo stochastic switches from phases of growth to shrinkage (catastrophe) and vice versa (rescue) [2]. During mitosis the less dynamic minus ends reside near the spindle poles while the fast growing plus-ends extend to the spindle equator and the cortex of the cell. A subset of spindle MTs is organized into distinct bundles (k-fibers) and attaches to the kinetochores, a multiprotein complex assembled on chromosomal centromeres.

The kinesin superfamily proteins (Kifs) share a common 360 amino acid (aa) sequence that is highly conserved throughout the eukaryotic phyla. This conserved globular domain, called the catalytic core, contains both a catalytic pocket for the hydrolysis of ATP and the binding site for MTs. The mechanical properties of kinesins are determined by the motor-domains (comprising the catalytic core and the adjacent neck region), whereas the stalk and tail domains can mediate dimerization and binding of cargo molecules, respectively. ATP hydrolysis mediates conformational changes in the catalytic core and neck region resulting in the movement of the motor along the MT lattice [3], [4], [5]. Members of the kinesin-13 family are exceptional in that they are immotile kinesins that utilize the energy of ATP hydrolysis to catalyze the depolymerization of MT ends [6]. Members of the Kinesin-8 family can be found in most eukaryotes ranging from fungi (KipB, *A. nidulans*; Kip3p, *S. cerevisiae*; klp5/6+, *S. pombe*), and Drosophila (Klp67A), to mammals (Kif18A). Loss of kinesin-8 activity results in hyperstable MTs and elongated spindles, accompanied by severe chromosome congression defects. *In vitro*, kinesin-8 proteins are slow plus-end directed motors and, as shown for the budding yeast orthologue, are characterized by a remarkably high processivity [7], [8], [9], [10], [11]. Upon accumulation at the plus-ends, Kip3p depolymerizes MTs in a length-dependent manner. According to the “antenna model”, the length dependent depolymerization originates from the high processivity of Kip3p which enables the motor once it lands on MTs to reach their plus-ends resulting in Kip3p levels at the tips of MTs that correlate with the length of MTs.

Upon entry into mitosis, Kif18A localizes to the lattice of spindle MTs from where it translocates to the plus-ends of kt-MTs during early metaphase [7]. Depletion of Kif18A causes severe chromosome congression defects and reduced tension on sister kinetochores resulting in the activation of the spindle assembly checkpoint (SAC) and, hence, in a mitotic delay. *In vitro* studies have shown that Kif18A like Kip3p depolymerizes MT in a length-dependent manner [7], [12]; an observation that has been recently challenged [13]. In depth live-cell studies revealed that loss of Kif18A increases the amplitude of chromosome oscillations whereas overexpression of Kif18A suppresses the movement of metaphase chromosomes suggesting that Kif18A negatively regulates chromosome oscillation in metaphase [8].

In this study, we investigated how Kif18A localizes to the plus-ends of kt-MTs. We found that a non-motor binding region site located at the C-terminus of Kif18A is essential for correct plus-end localization in mitosis. In HeLa-cells, Kif18A lacking the C-proximal 121 residues decorated the lattice of spindle MTs but did not display prominent plus-end localization. In line with its inability to localize correctly, tail-less Kif18A failed to rescue spindle length and chromosome alignment in cells depleted of endogenous Kif18A. The complementary C-tail fragment accumulated at spindle poles where it partially co-localized with pericentrin. *In vitro* studies demonstrated that the C-terminus of Kif18A can bind directly to MTs. Intriguingly, single molecule measurements revealed that Kif18A lacking this additional MT binding site displays shorter run lengths and higher velocity compared to full-length protein. Thus, the C-proximal tail of Kif18A contributes to its processivity required for its plus-end localization and, hence, its function in mitosis.

## Results

**Figure 1:**
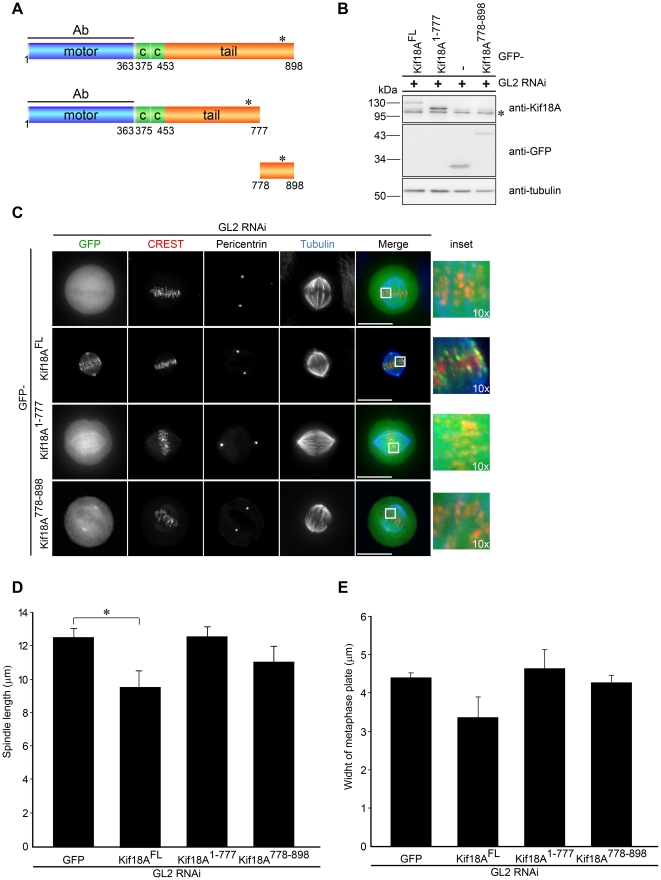
The C-terminus of Kif18A is essential for its plus-end accumulation on kt-MTs. (**A**) Schematic representation of Kif18A sequences used as GFP-tagged constructs in this study. The region for antibody (Ab) production is indicated as a line. Asterisk indicates a functional nuclear localization signal (NLS). CC indicates a putative coiled-coil. (**B**) Extracts from HeLa-cells transfected with a small interfering RNA (siRNA) duplex targeting GL2 (control) and Kif18A siRNA resistant GFP-constructs were probed by immunoblotting. After cutting the membrane in halve, GFP-Kif18A^FL^ and -Kif18A^1-777^ were detected with antibodies raised against the N-terminus of Kif18A (upper panel) and GFP-Kif18A^778-898^ with antibodies against the GFP tag (lower panel). An anti-α-tubulin immunoblot serves as loading control. (**C**) Localization of transiently overexpressed GFP, GFP-Kif18A^FL^, GFP-Kif18A^1-777^ and GFP-Kif18A^778-898^ during metaphase in HeLa-cells treated with GL2 control siRNA determined by immunofluorescence. HeLa-cells were stained with CREST antisera (to mark kinetochores), anti α-tubulin (blue), anti-pericentrin. Kif18A was visualized by GFP-tag. The scale bar is 15 µm. All images are z-projections of deconvolved 3D stacks. The merge image represents GFP, CREST and tubulin. (**D**) Quantification of pole to pole distance. The bar represents the average of 3 independent experiments (n = 10-20 cells). (**E**) Quantification of chromosome alignment. The bar represents the average of 3 independent experiments (n = 10-20 cells). Asterisk in (D) indicates statistical difference (p = 0.018; significance level 0.05; t test, 2-tailed). Errorbars in (D) and (E) indicate SD. Asterisk in **(B)** indicate endogenous Kif18A.

Sequence analyses of full-length Kif18A (898 amino acids; aa) using the human protein reference database (http://www.hprd.org/) revealed an N-proximal motor domain consisting of a catalytic motor and neck (aa 9-363), a centrally positioned putative coiled-coil region (aa 375-454), and a C-terminal tail (aa 453-898) that includes a functional nuclear localization signal (NLS; aa 828-832) [13] (Fig. 1A). To investigate which domains of Kif18A contribute to its localization in mitosis, we expressed Kif18A fragments fused at their amino-terminus to green fluorescent protein (GFP) in HeLa-cells. In a first series of experiments, we analyzed the mitotic localization of GFP-Kif18A variants in control RNA-interference (RNAi) HeLa-cells transfected with short interfering RNA-oligonucleotides (siRNAs) targeting luciferase (GL2). Consistent with previous studies [7], [8] GFP-tagged full-length Kif18A (Kif18A^FL^) like the endogenous protein localized to the tips of spindle microtubules (Fig. 1C). Co-staining with anti-kinetochore antibodies (CREST serum [14]) confirmed that GFP-Kif18A^FL^ accumulated at the plus-ends of kt-MTs in GL2-RNAi HeLa-cells (Fig. 1C). Reportedly, motor-dead Kif18A localizes to the lattice rather than the tips of spindle MTs [8] implicating that Kif18A upon binding utilizes its plus-end directed motility to accumulate at the tips of MTs. Notably, however, we observed that a motor-only Kif18A variant, GFP-Kif18A^1-367^ localized diffusely with an accumulation on the mitotic spindle (Fig. S1). Thus, Kif18A's motor-activity while being required is not sufficient for correct localization. To identify the additional region of Kif18A critical for its plus-end localization, three carboxy-terminal truncations of Kif18A were generated: aa^1-467^, aa^1-526^, and aa^1-777^ all of which comprised the motor-domain, coiled coil region implicated in dimerization, and C-terminal extensions of increasing length. Intriguingly, we observed that even GFP-Kif18A^1-777^ when expressed in control GL2-RNAi HeLa-cells did not correctly localize as it primarily decorated the MT lattice with a slight accumulation at MT ends (Fig. 1C and S1) suggesting that the carboxy-terminal 121 residues of Kif18A are critical for proper plus-end localization. When we analyzed the complementary fragment, GFP-Kif18A^778-898^, we observed no significant colocalization with the CREST signal but an accumulation of GFP-Kif18A^778-898^ at spindle poles where it partially colocalized with pericentrin (Fig. 1C) indicating that the C-terminal tail by itself does not localize to the plus-ends of kt-MTs. Efficient expression of GFP-Kif18A variants was confirmed by Western blot analyses where cells were lysed and the SDS-PAGE immunoblot was cut in halve to detect GFP-Kif18A^FL^ and -Kif18A^1-777^ with antibodies raised against the N-terminus of Kif18A (Fig. 1B, upper panel) and GFP-Kif18A^778-898^ with antibodies against the GFP tag (Fig. 1B, middle panel). In summary, these data suggest that both the motor-activity and the C-terminal tail are required for the correct plus-end accumulation of Kif18A at kt-MTs.

The overexpression of Klp67A induces spindle shortening [15] suggesting that spindle length is sensitive to increased kinesin-8 activity. In line with this finding, we observed that the pole-to-pole distance - using pericentrin as marker - was significantly decreased in HeLa-cells expressing GFP-Kif18A^FL^ compared to GFP-only expressing cells: 9,58 µm ± 0,98 µm and 12,51 µm ± 0,55 µm in GL2-RNAi HeLa-cells expressing GFP-Kif18A^FL^ and GFP-only, respectively (Fig. 1D). Consistent with their failure to localize correctly, neither GFP-Kif18A^1-777^ nor the complementary C-terminal fragment, GFP-Kif18A^778-898^ had a significant effect on spindle length (Fig. 1D). Reportedly, the width of the metaphase plate decreases as mammalian cells progress towards anaphase by the Kif18A-dependent suppression of chromosome oscillations [16]. Indeed, we observed that full-length Kif18A but neither of the two truncation constructs nor GFP-only when overexpressed in GL2-RNAi cells caused a decrease in the width of the metaphase plate (Fig. 1E). In summary, this set of experiments revealed that the motor-domain and the C-terminus of Kif18A are both important but not by themselves sufficient for proper localization to the plus-ends of kt-MTs and for regulating mitotic spindle length and chromosome alignment at the metaphase plate.

**Figure 2:**
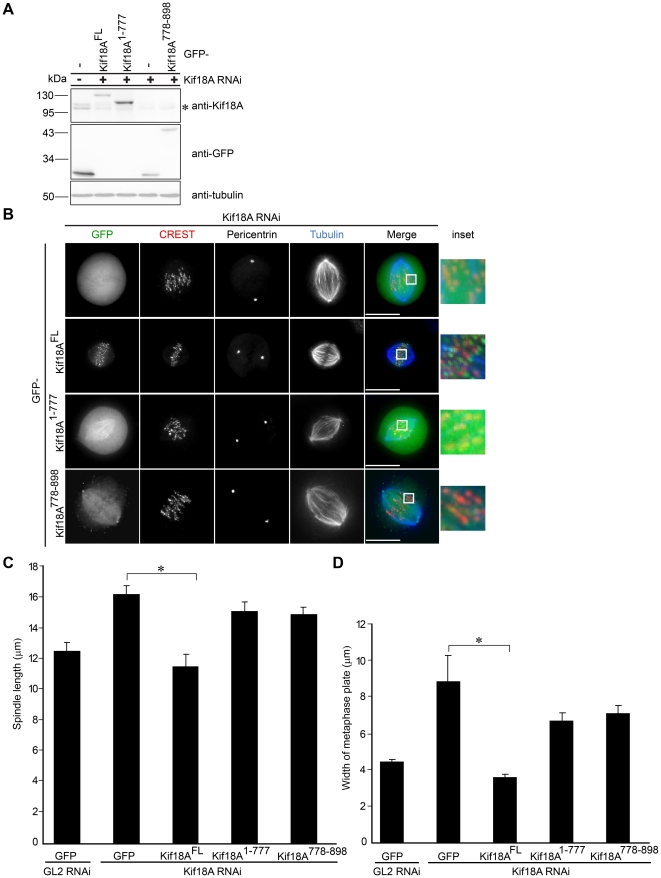
Kif18A lacking its C-terminus cannot complement loss of endogenous Kif18A. (**A**) Extracts from HeLa-cells transfected with a small interfering RNA (siRNA) duplex targeted to GL2 (control) or Kif18A and Kif18A siRNA resistant GFP-constructs were probed by immunoblotting with GFP and Kif18A antibodies. An anti-α-tubulin immunoblot served as loading control. (**B**) Localization of transiently overexpressed Kif18A siRNA resistant GFP, GFP-Kif18A^FL^, GFP-Kif18A^1-777^ and GFP-Kif18A^778-898^ during metaphase in HeLa-cells treated with Kif18A siRNA determined by immunofluorescence. HeLa-cells were stained with CREST antisera (to mark kinetochores; red), anti α-tubulin (blue), anti-pericentrin. Kif18A was visualized by GFP-tag. The scale bar is 15 µm. All images are z-projections of deconvolved 3D stacks. The merge image represents GFP, CREST and tubulin. (**C**) Quantification of pole-to-pole distance. The bar represents the average of 3 independent experiments (n = 30-40 cells). (**D**) Quantification of chromosome alignment. The bar represents the average of 3 independent experiments (n = 30-40 cells). Asterisk indicates statistical difference (p = 0.0017 in (C) and p = 0.022 (D) significance level 0.05; t test, 2-tailed). Errorbars in (C) and (D) indicate SD. Asterisk in (A) indicates endogenous Kif18A.

Loss of Kif18A induces spindle lengthening and severe defects in chromosome alignment [7], [8]. To test if the different Kif18A truncations can functionally rescue loss of Kif18A, we expressed them as GFP-fusions in HeLa-cells depleted of endogenous Kif18A. mRNAs used for the expression of the different Kif18A fragments contained five silent mutations in the target sequence of the Kif18A siRNA duplexes to allow the expression of the rescue constructs in Kif18A-depleted cells. To enrich for mitotic cells, HeLa-cells were released from a single thymidine block for nine hours followed by an one hour incubation with the proteasome inhibitor MG132. The efficient depletion of endogenous Kif18A and expression of ectopic GFP-Kif18A variants were confirmed by immunoblotting for Kif18A and GFP as described before (Fig. 2A). First, we analyzed the localization of the different GFP-Kif18A variants in Kif18A-RNAi HeLa-cells. Immunofluorescence analyses revealed that GFP-Kif18A^FL^ was clearly enriched at the plus-ends of kt-MTs as indicated by the partial co-localization with the CREST signal, whereas GFP-Kif18A^1-777^ predominantly decorated the lattice of spindle MTs (Fig. 2B). Like in control-depleted cells, GFP-Kif18A^778-898^ was absent from the plus-end of kt-MTs but enriched at spindle poles where it partially co-localized with pericentrin (Fig. 2B). Next, we investigated the functionality of the different Kif18A variants. Consistent with previous observations [7], [8], loss of Kif18A induced spindle lengthening from 12,51 µm ± 0,55 µm (GL2-RNAi cells expressing GFP-only, Fig. 2B and C) to 16,24 µm ± 0,57 µm (Kif18A-RNAi cells expressing GFP-only, Fig. 2B and C). Expression of GFP-Kif18A^FL^ efficiently restored spindle length in Kif18A-depleted cells to 11,50 µm ± 0,80 µm (Fig. 2B and C). Again, both GFP-Kif18A^1-777^ and -Kif18A^778-898^ did not significantly restore wildtype pole-to-pole distance in Kif18A-RNAi cells: 15,11 µm ± 0,62 µm (GFP-Kif18A^1-777^); 14,98 µm ± 0,44 µm (GFP-Kif18A^778-898^) (Fig. 2B and C). Consistent with its key function in chromosome congression [7], [17] Kif18A-depleted cells displayed unaligned chromosomes scattered between the two poles of the bipolar spindle. Quantification revealed that the width of the metaphase plate increased from 4,40 µm ± 0,13 µm in control-depleted GFP-expressing cells (Fig. 2D) to 8,80 µm ± 1,44 µm in cells depleted of Kif18A and expressing the GFP-only control (Fig. 2B and D). As expected, expression of GFP-Kif18A^FL^ restored chromosome alignment in Kif18A depleted cells as indicated by a metaphase plate width of 3.56 µm ± 0.18 µm (Fig. 2B and D). In line with their effect on spindle length, the expression of neither GFP-Kif18A^1-777^ nor -Kif18A^778-898^ efficiently rescued the alignment of chromosomes at the spindle equator in HeLa-cells depleted of endogenous Kif18A: 6,65 µm ± 0,46 µm and 7,07 µm ± 0,43 µm width of the metaphase plate in Kif18A-RNAi cells expressing GFP-Kif18A^1-777^ and -Kif18A^778-898^, respectively (Fig. 2B and D).

**Figure 3:**
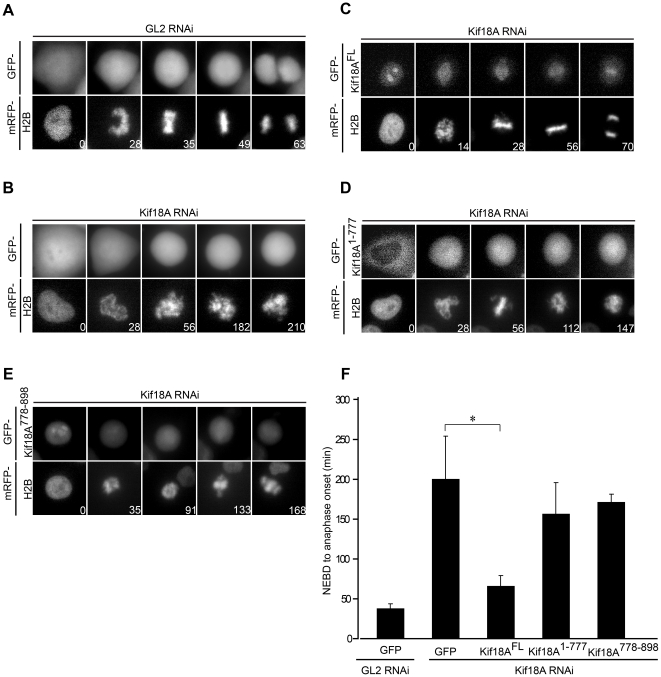
Cells expressing Kif18A lacking its C-terminus are delayed in anaphase onset. (A) – (E) Selected live-cell stills of HeLa-cells stably expressing H2B-mRFP transfected with GL2 or Kif18A siRNA oligos and indicated GFP-tagged constructs. (F) Quantitative analyses of the time-lapse microscopy as shown in (A-E) documenting the average time required from NEBD to anaphase onset in control or Kif18A depleted cells expressing the indicated Kif18A siRNA resistant GFP-constructs (n = 50–100) from 3 independent experiments. Numbers in (A) to (E) represent time in min. Error bars in (F) indicate SD. Asterisk indicates statistical difference (p = 0.044 in (F) significance level 0.05; t test, 2-tailed).

We then used live-cell microscopy analyses to study the functionality of tail-less (GFP-Kif18A^1-777^) and tail-only Kif18A (GFP-Kif18A^778-898^). Our previous studies revealed that Kif18A depletion results in SAC activation and, thus, in a mitotic delay [7]. When we performed time-lapse microscopy with HeLa-cells expressing histone H2B fused to monomeric red fluorescent protein (mRFP) we observed that the depletion of Kif18A increased the length of time from nuclear envelope breakdown (NEBD) to anaphase from 37.07 min ± 6.31 min (GL2-RNAi cells expressing GFP-only) to 199.56 min ± 54.81 min (Kif18A-RNAi cells expressing GFP-only) (Fig. 3A, B, and F, Movies S1, S2, S3, S4,). This delay in anaphase onset was efficiently rescued by the expression of GFP-Kif18A^FL^ (time form NEBD to anaphase onset: 65.82 min ± 13.73 min, Fig. 3C and F, Movie S5, S6). In line with their inability to efficiently rescue spindle defects, expression of neither GFP-Kif18A^1-777^ nor GFP-Kif18A^778-898^ had a significant effect on the time elapsing from NEBD to anaphase onset in Kif18A-RNAi cells: 156.33 min ± 39.75 min and 172.16 min ± 11.43 min in cells expressing GFP-Kif18A^1-777^ and GFP-Kif18A^778-898^, respectively (Fig. 3D, E, and F, Movies S7, S8, S9, S10). Analyses of the H2B-mRFP movies revealed that cells expressing tail-less or tail-less GFP-Kif18A aligned only a fraction of the chromosomes at the spindle equator and the metaphase plate-like structures that formed were often not robust but disappeared over time (Fig. 3D and E). In summary, our studies revealed that the C-proximal 121 residues of Kif18A are critical for the mitotic function of Kif18A.

**Figure 4:**
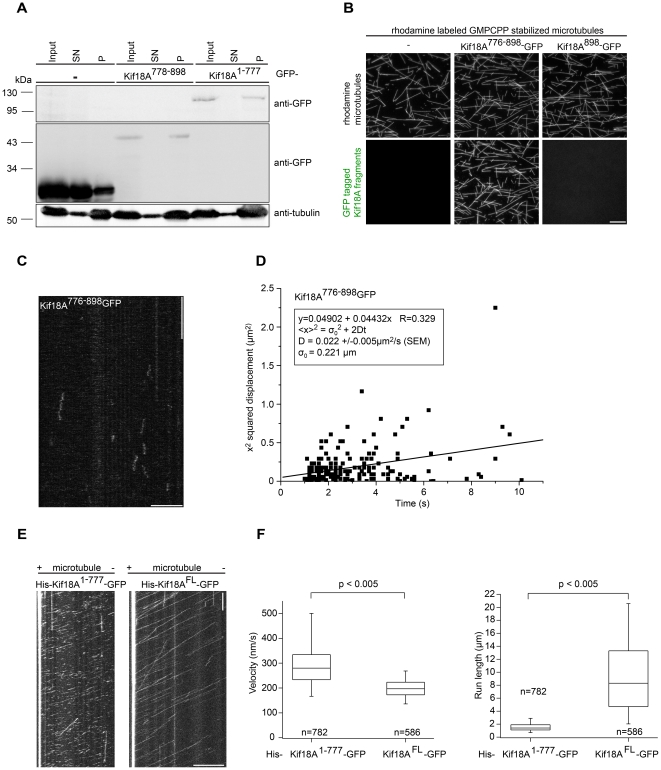
The high processivity of Kif18A depends on its C-proximal tail. (**A**) Binding of GFP-Kif18A^1-777^ and GFP-Kif18A^778-898^ to mitotic microtubules revealed by a co-sedimentation assay. Input (I), supernatant (SN) and Pellet (P) fractions were probed by immunoblotting with GFP and tubulin antibodies. (**B**) Rhodamine-labeled GMPCPP stabilized microtubules are immobilized in a flow channel with anti-tubulin antibodies and imaged with TRITC epifluorescence microscopy (upper panel). As revealed by 488 nm TIRF microscopy (lower panel) Kif18A^776-898^–GFP (0.6 µM) decorates microtubules, whereas Kif18A^898^–GFP (0.6 µM) does not. (**C**) Kymograph showing diffusing Kif18A^776-898^-GFP on microtubules imaged by TIRF microscopy. Horizontal scale bar indicates 5 µm, vertical scale bar indicates 10 s. (**D**) Linear least square fit of squared displacement of Kif18A^776-898^-GFP over diffusion time allows calculation of the diffusion constant. Only traces of molecules diffusing for at least 1s were included. (**E**) Kymographs showing movement of single His-Kif18A^1-777^-GFP molecules (left panel) and His-Kif18A^FL^-GFP (right panel) along a microtubule as imaged by TIRF microscopy. Horizontal scale bars indicate 10 µm, vertical scale bar indicates 1 min. Plus (+) and minus (–) symbols above the image show the polarity of the microtubule. (**F**) Analysis of single molecule measurements of the velocity (left panel) and run lengths, the distance travelled before dissociation (right panel), of His-Kif18A^1-777^-GFP and His-Kif18A^FL^-GFP. Statistical difference was determined by a 2-tailed t test. Box plots show 5th, 25th, 50th, 75th and 95th percentiles.

Yeast kinesin-8 family displays an exceptionally high processivity which allows the motors, once they bind to the lattice of spindle MTs, to reach the plus-ends and accumulate there [11], [18]. Our observation that tail-less Kif18A decorates the lattice but fails to accumulate at MT plus-ends suggests that the C-terminus of the protein might contribute to the high processivity of the motor. Furthermore, given that GFP-Kif18A^778-898^ displayed a centrosome proximal spindle localization it is tempting to speculate that the C-terminal tail of Kif18A contributes to its processivity by providing an additional MT binding site. To test whether Kif18A^778-898^ can bind to MTs, we prepared lysate of mitotic HeLa-cells expressing GFP-Kif18A^778-898^ and -Kif18A^1-777^ or the GFP-control and induced the polymerization of stable MTs by the addition of 1 mM GTP and 10 µM taxol. After a 30-minute incubation at room temperature, MTs were pelleted by high-speed centrifugation and the pellet (P) and supernatant (SN) fractions were analyzed by immunoblotting. As expected, GFP-Kif18A^1-777^ comprising the motor MT binding domain co-sedimented with MTs in the pellet fraction (Fig. 4A). Intriguingly, GFP immunoblot analyses detected GFP-Kif18A^778-898^ primarily in the MT pellet fraction (Fig. 4A). The co-sedimentation with MTs was mediated by the C-terminal tail of Kif18A as GFP by itself was primarily detected in the supernatant fraction (Fig. 4A). To confirm that the C-proximal tail of Kif18A binds directly to MTs, we performed *in vitro* MT binding assays. To this end, we expressed Kif18A (residues 776-898) and as control the last residue of Kif18A (residue 898) fused at their amino- and carboxy-terminus to maltose-binding protein (MBP) and GFP, respectively. Recombinant proteins bound to the amylose resin were eluted by cleaving off the MBP tag using human rhinovirus 3C protease (Fig. S1C). Rhodamine-labeled MTs were immobilized in passivated flow cells *via* surface-adsorbed anti-tubulin antibodies and imaged using epifluorescence microscopy (Fig. 4B, upper panel). Intriguingly, after infusion of Kif18A^776-898^-GFP we observed the efficient decoration of MTs with the C-terminal tail of Kif18A (Fig. 4B, lower panel). The binding of the Kif18A-fusion protein to MTs was mediated by the Kif18A moiety as the GFP-control did not show any colocalization (Fig. 4B, lower panel). Thus, the C-proximal tail of Kif18A possesses an additional MT binding site. Interestingly, the proximal tail was not immobile on the microtubule lattice. Using total-internal reflection-fluorescence (TIRF) microscopy, we could demonstrate that single molecules of Kif18A^776-898^-GFP underwent randomly-directed motion on the lattice (Fig. 4C) with a diffusion coefficient of 0.022 ± 0.005 µm^2^/s (Fig. 4D).

Finally, we investigated if this non-motor MT binding site affects the motility of Kif18A. For these experiments, we purified full-length Kif18A^FL^-GFP and tail-less Kif18A^1-777^-GFP fused at their N-termini to a six-histidine (His) tag from insect cells Fig. S1D). The movement of individual molecules along the lattice of MTs was visualized by TIRF-microscopy (Fig. 4E, Movies S11, S12). Tail-less Kif18A moved significantly faster than full-length protein: 299 nm/s (SD  =  109 nm/s, *N* = 782) and 199 nm/s (SD  =  39 nm/s, *N* = 586) for His-Kif18A^1-777^-GFP and His-Kif18A^FL^-GFP, respectively (Fig. 4F, left panel). Tail-less Kif18A was dramatically less processive than the full-length protein. The average run length of motors that dissociated from the lattice was 1.6 µm (SD  =  0.7 µm. N = 782) for His-Kif18A^1-777^-GFP, much smaller than 9.4 µm (SD  =  5.7 µm, N = 586) for His-Kif18A^FL^-GFP (Fig. 4F, right panel). Because of its high processivity, many of the full-length motors reached the MT end without dissociating and were not counted in this analysis; this leads to an underestimate of the run length. An unbiased estimated of the true mean run length was obtained by dividing the total run length of all the motors moving on the lattice (whether they dissociated or not), by the number of total number of dissociations. This gives a mean run length of 12.9 µm (7554 µm/586 dissociations). Notably, when we performed bleaching experiments in a TIRF streaming mode, we observed only one and two step bleaching events for both His-Kif18A^1-777^-GFP and His-Kif18A^FL^-GFP (Fig. S1E, F, and G) indicating that both proteins form stable dimers. To further corroborate this finding, we performed co-immunoprecipitation experiments from cells expressing GFP- and Myc-tagged Kif18A^1-777^. As shown in figure S1B, Myc-Kif18A^1-777^ efficiently co-precipitated with GFP-tagged Kif18A^1-777^ demonstrating that tail-less Kif18A is able to form dimers in cells. Thus, in line with the fact that Kif18A^1-777^ contains both predicted coiled-coil motifs these data reveal that tail-less Kif18A like the full-length protein forms a stable dimer suggesting that defects in dimer formation does not account for the observed decrease in processivity of Kif18A^1-777^.

In summary, our single molecule velocity measurement demonstrate that Kif18A like its yeast orthologue [11], [18] is a highly processive plus-end directed motor protein and, furthermore, that the C-terminus of Kif18A possesses a non-motor MT binding site which is critical for the high processivity of the motor.

## Discussion

In mammalian cells, the faithful distribution of the genome depends on the activity of the kinesin-8 protein Kif18A. Loss of Kif18A results in lengthening of mitotic spindles and severe chromosome congression defects and consequently, in a SAC-dependent mitotic delay. Critical for the proper function of Kif18A is its plus-end localization to kt-MTs. During progression from prometaphase to metaphase, Kif18A translocates from spindle MTs to the tips of kt-MTs where it accumulates in proximity to the outer kinetochore protein Hec1 [7]. The plus-end accumulation of Kif18A depends on its motor activity as a mutant form unable to translocate along MTs, decorates the MT lattice but fails to localize to MT plus-ends [8].

We addressed here the question of how Kif18A translocates to the plus-ends of MTs. We found that the motor domain of Kif18A while being essential for plus-end accumulation is not sufficient for proper localization. Specifically, we revealed that GFP-tagged Kif18A lacking its C-proximal 121 residues primarily decorated the lattice of spindle MTs but did not display prominent plus-end localization. Importantly, this Kif18A truncation failed to localize correctly not only in control-RNAi cells but also in cells depleted of endogenous Kif18A excluding the possibility that competition with wildtype protein accounts for the observed localization defects. In search for the function of the C-terminus, we identified a non-motor MT binding site. Using MT pull down assays from cell lysates as well as in vitro MT decoration assays with purified components, we could demonstrate that the C-proximal 121 residues of Kif18A are competent in directly binding to MTs. Notably, in line with our results, the existence of an additional MT binding site in the C-terminal tail of Kif18A and yeast Kip3p was recently also reported by others [19], [20], [21]. How does this additional MT binding site contribute to plus-end localization of kinesin-8 motors? Our TIRF-analyses revealed that Kif18A lacking its C-proximal MT binding site had a dramatically shorter run length but increased velocity compared to full-length protein. As shown by our photobleaching experiments, both proteins full-length and tail-less His-Kif18A-GFP displayed only one step and two step bleaching events implicating that Kif18A^1-777^ like the full-length protein forms stable dimers. Furthermore, the C-terminal tail was not immobile on MTs but rather displayed randomly-directed motion on the MT lattice. Thus, our data suggest that the C-terminus of Kif18A comprising the additional MT binding site contributes to the high processivity of the motor by acting as a tether preventing the dissociation of Kif18A from the MT lattice; for example, the tail may concatenate several shorter runs, like those seen for the truncated protein, into the longer runs seen for the full-length protein. Notably, tail-less Kif18A/Kip3p distinguishes itself from full-length protein not only in reduced processivity but also in reduced pausing time at the plus-ends of MTs [19], [20] suggesting that the additional MT binding site is also required for efficient MT plus-end binding.

Our observation that Kif18A lacking its C-proximal tail did neither rescue spindle length nor chromosome alignment in Kif18A-depleted cells is in line with the antenna model postulating that the MT dynamics regulatory function of kinesin-8 proteins depends on their MT length-dependent flux to the plus ends of MTs. As its depolymerization activity is weak compared to kinesin-13 [22], arrival of a sufficiently high number of kinesin-8 proteins would be required to mediate plus-end specific depolymerization. Intriguingly, the velocity of 199 nm/s of full-length Kif18A as determined by our TIRF-experiments exactly matches the minimal velocity of 200 nm/s predicted by computational modeling to be required to ensure sufficient MT plus-end localization of Kif18A [21].

Notably, *Drosophila* Klp67A displays a different mechanism of localization in that it is already recruited to prophase kinetochores in a MT-independent manner [9]. Upon attachment of kinetochores by MTs, Klp67A utilizes its motor activity to regulate chromosome alignment and spindle length. Intriguingly, while Klp67A as a core kinetochore component is clearly distinct in its kt-MT plus-end targeting mechanism to Kif18A, it has been shown that the Klp67A tail is essential for spindle association [9]. Thus, the existence of a C-proximal MT binding site and its function in localization might be a common feature of kinesin-8 proteins. There is increasing evidence that the activities of kinesins are regulated by posttranslational modifications of the tail-domain [23], [24], [25]. Therefore, it is tempting to speculate that the C-terminus of Kif18A might be under posttranslational control providing an elegant mechanism to fine-tune Kif18A activity in cells. Clearly, future studies are required to investigate this exciting possibility.

## Materials and Methods

### Constructs

All human Kif18A (NM_031217) constructs were PCR amplified from human testis cDNA (Invitrogen) with the following primer pairs: (aa 1–898): 5′ primer: ATTAGG-CCGGCCAATGTCTGTCACTGAGGAAG; 3′ primer: ATTAGGCGCGCCCTTAGATTT-CCTTTTGAAATATTTC; huKif18A (aa 1–777): 5′ primer: ATTAGGCCGGCCAATGT-CTGTCACTGAGGAAG; 3′ Primer: GGCGCGCCGTTACTTGATGTCTTCACATATAG; huKif18A (aa 778-898): 5′ primer: ATTAGGCCGGCCGATGAGCTCGAAGTGTAA-ATTACCCG; 3′ Primer: ATTAGGCGCGCCCTTAGATTTCCTTTTGAAATATTTC. The following primers were used to generate silent mutations for rendering pCS2-GFP-Kif18A^FL^ and pCS2-GFP-Kif18A^1-777^ resistant to the Kif18A siRNA oligo: primer pair 1: TACCAACAACAGTGCCACAAGCAAATAGAAATGATGTG; CACATCATTTCTATTTGCTTGTGGCACTGTTGTTGGTA; Primer pair 2: TGAACTTAAATCATTC-TATCAGCAACAGTGCCACAAGCAA; TTGCTTGTGGCACTGTTGCTGATAGAATG-ATTTAAGTTCA; Primer pair 3: TAAATCATTCTATCAGCAGCAATGCCACAAGCAA-ATAGAA; TTCTATTTGCTTGTGGCATTGCTGCTGATAGAATGATTTA; Primerpair4: ATTCTATCAGCAGCAATGTCACAAGCAAATAGAAATG; CATTTCTATTTGCTTGTGACATTGCTGCTGATAGAAT. The amplified cDNAs were cloned into pCS2-GFP plasmids harboring engineered FseI and AscI restriction sites at the 5′ and 3′ end. For single molecule studies Kif18A(aa 1-898) and Kif18A (aa 1-777) were cloned into a modified FastBacM13 vector carrying an n-terminal 6xHis tag and a c-terminal GFP-Avitag. Kif18A (aa 776-898)-GFP and Kif18A (aa 898)-GFP were cloned from the FastBacM13 into a modified bacterial expression vector petM42 (n-terminal maltose binding protein with 3-C protease cleavage side).

### Protein Expression

The BAC-TO-BAC expression system (Invitrogen, Paisley, UK) was used to express 6xHis-Kif18A (aa 1-898)-GFP-Avi and 6xHis-Kif18A (aa 1-777)-GFP-Avi in insect cells SF+ (obtained from protein science). Cells were cultured in serum free SF900II Medium (Invitrogen). Protein Purification was performed as described in [7]. Kif18A^776-898^–GFP and Kif18A^898^–GFP were expressed in BL21(DE3)-T1R competent Escherichia coli (Sigma B2935) transformed with pRARE (Novagen, # 70954). Expression was induced at OD 0.6 with 0.5 mM IPTG over night at 18°C. Proteins were extracted with B-PER (Thermo Fischer) according to manufacturer's protocol. The extract was cleared by centrifugation at 10 min at 10,000 x g. The supernatant was incubated with Amylose resin (NEB) for 2 h at 4°C and the resin was washed four times with 3C-buffer (50 mM TrisHCl pH 7.4, 150 mM NaCl, 1 mM EDTA, 1 mM DTT, 0.01% Tween20). 3C-Protease (gift from David Drechsel, MPI-CBG) was added to cleave Kif18A C-terminus-GFP from its N-terminal maltose binding protein. Resin beads where pelleted and supernatant was snap frozen in liquid nitrogen.

### Antibody Production

GST-tagged huKif18A (aa 1-367) was purified as described in [7] and was used for immunizing rabbits. Antibodies against huKif18A (aa 1-367) were purified with MBP-huKif18A (aa 1-367) coupled to N-hydroxysuccinimid activated sepharose beads (Amersham Pharmacia) as affinity matrix.

### Antibodies

The following primary antibodies were used: affinity-purified anti-huKif18A 1∶400 (western-blot analyses, WB); polyclonal anti-Pericentrin (abcam,no.4448) 1∶000 (immunofluorescence,IF); anti-tubulin (DM1alpha,Sigma,No.T6199): 1∶1000 (WB,IF); CREST serum (Immunovision) 1∶3000 (IF), polyclonal anti-GFP (abcam,No.290) (1∶1000) (WB). The following secondary antibodies were used: Horseradish-peroxidase-conjugated anti-mouse or anti-rabbit (Dianova) 1∶3000 (WB); goat anti-rabbit alexa fluor 568 (Invitrogen) (1∶1000), goat anti-human alexa fluor 647 (Invitrogen,No. A21445) (1∶500), goat anti-mouse alexa fluor 350 (Invitrogen,No.11045) (1∶50).

### Cell Culture

All cell lines were grown in Dulbecco's modified Eagle medium (Invitrogen) with 10% fetal-bovine serum (Invitrogen) and 1 U penicillin-streptomycin (Invitrogen) at 37°C in a humid atmosphere with 5% CO_2_. For immunofluorescence experiments, we used HeLa-cells (same clone as used in [7]). For live cell studies we used HeLa-cells stably expressing H2BmRFP (same clone as used in [26]).

### siRNA Experiments

RNAi transfections were performed at 75 nM final concentration using Oligofectamine (invitrogen) following the manufacturer's protocol. For experiments performed on coverslips (Marienfeld) in 6-well plates, cells were plated 24h prior to transfection. The following siRNA oligonucleotides were used: Luciferase GL2 target sequence: (5′-AACGUACGC-GGAAUACUUCGA-3′) (Dharmacon Research). Kif18A target sequence: 5′-AACCAA-CAACAGUGCCAUAAA-3′ (Dharmacon Research). Cells were fixed and processed for immunofluorescence or lysed for western-blot analysis 48 hr after transfections.

### Transfection Experiments and cell extracts

HeLa-cells were seeded on coverslips at 4*10^4^ cells/ml in a six-well plate 24 hr before transfection. HeLa-cells were transfected with the following plasmids: GFP, GFP-Kif18A^FL^, GFP-Kif18A^1-777^ and GFP-Kif18A^778-898^ using Fugene6 in accordance with the manufacturer's protocol (Roche). After 10 hours medium was removed and the siRNA transfection-mix was dropped carefully. After 20 hours Thymidine (Sigma) was added to a final concentration of 2 mM for 18 hours. Cells were released and after 9 hours MG132 (Sigma) was added to a final concentration of 20 µM for 1 hour. Cells were then either lysed in sample buffer or processed for immunofluorescence as described. For co-immunoprecipitations, 293T cells were harvested and lysed with lysisbuffer (0.5% Triton-X100, 30 mg/ml DNase, 30 µg/ml RNase 1 mM DTT, 20 mM ß-glycerophosphat, 20 mM NaF, 0,3 mM NaV, 300 mM Nacl, 25 mM Tris pH 7.4, 5 mM EDTA and protease inhibitors. The antibodies for GFP were coupled to Protein G beads (Pierce), incubated with the cleared extract and bound proteins were precipitated.

### Immunoblotting

Cell extracts were boiled in sample buffer and resolved by SDS-polyacrylamide gel electrophoresis (SDS-PAGE). Proteins were transferred to Nitrocellulose transfer membrane (Schleicher and Schuell) and then blocked with 5% slim-milk powder in PBS + 0.1% Tween-20 solution. Blots were developed by an ECL westernblotting detection system and visualized with a CCD camera (Raytest-1000, Fujifilm).

### Fixation, Fluorescence Microscopy, and Live-Cell Imaging

Cells were fixed for 12 min in fixation buffer (200 mM PIPES at pH 6.8, 20 mM EGTA, 2 mM MgCl_2_, 0.4% Triton X-100, and 4% formaldehyde). Samples were washed with TBS + 0.1% Triton X-100 (TBST) and incubated for 1 hr in TBST + 2% bovine-serum albumin (Sigma). Antibodies were incubated for 1 hr at RT. Washed samples were embedded in mounting media (20 mM Tris-HCl [pH 8.8], 0.5% phenylendiamine, and 90% glycerol). High resolution images as shown in Figures 1, 2 and Figure S1 were taken as z-stacks on a DeltaVision Core System using a Nikon PlanApo N 60x, N.A. 1.42, W.D. 0.15 oil objective (Olympus). A CoolSnap HQ digital camera was used. For live-cell studies, HeLa-cells stably expressing H2BmRFP were treated with GL2 or Kif18A-siRNA oligos and transferred to CO_2_-independent media before recording on a Zeiss Observer.Z1 inverted microscope with a heating chamber at 37°C on 12-well plate using a Plan-Neofluar 40 x, N.A. 0.6, (Zeiss). Images were taken every 7 min. and analyzed with the Metamorph software.

### Software and image processing

High-resolution images were acquired as *z*-stacks on a DeltaVision Core System and deconvolved using softWorx 4.0 software (Applied Precision). Projections were generated using the ‘quick projection’ function using the maximal intensity in the stack for each pixel. Images of fixed samples were exported as 8-bit grayscale tiff files for every channel. Figures were assembled in Adobe Photoshop CS2 and Adobe Illustrator CS2 without any further adjustments to microscopy images. Live-cell recordings were analyzed and processed with Metamorph software (Molecular Devices). Images in Figure 4C and 4D were taken with Metamorph and kymographs where derived from microtubules longer than 20 µm. Individual motors that travelled further than 400 nm and dissociated before reaching the MT end were measured with Fiji (http://fiji.sc/wiki/index.php/Fiji) and the velocities and run lengths calculated. These criteria led to small biases in the measurements of mean run length: for the more processive full-length kinesin the mean run length was underestimated (because motors that travelled to the end without dissociating were not counted in the analysis), whereas for the less processive truncated kinesin the mean run length was overestimated (because runs shorter than 400 nm were missed in the analysis). These biases lead to an underestimate of the difference in processivity between the full-length and truncated kinesins. For a less biased estimate of the mean run length of full length Kif18A, all run distances in the kymographs where summed and divided by the number of dissociations. To analyze diffusion of Kif18A (aa 776-898)-GFP, the times and distances that particles travelled between landing and dissociating from the microtubule lattice were measured. Runs shorter than 1 s were excluded. The squared displacement was plotted over time and fit to a line using least-squares. Data were analyzed in Igor Pro (Wavemetrics) and Origin (Originlab).

### Microtubule sedimentation experiments

HeLa-cells were transfected with plasmids encoding for GFP, GFP-Kif18A^1-777^ and GFPKif18A^778-898^ as described above. After 24 hours Thymidine (Sigma) was added to a final concentration of 2 mM for 20 hours. Afterwards cells were released and VS-83 (synthesized by Vasiliki Sarli [27] and was a kind gift of the lab of Athanassios Giannis) was added at a final concentration of 30 µM. Mitotic cells were collected by “mitotic shake off”, washed once in PBS and lysed in BRB80 supplemented with 0,5% TritonX-100, 115 mM KCl, 300 mM Sucrose, 5 mM MgCl_2_, 1 mM EGTA, 20 mM ß-glycerophosphat, 20 mM NaF, 0,3 mM Na_3_VO_4,_ 1 mM DTT, and protease inhibitors (Roche) before centrifugation at 14.000 rpm at 4°C for 5 min. The clarified supernatant was then centrifuged at 90.000 rpm (TLA100, Beckman) at 4°C for 40 minutes. 1 mM DTT, 1 mM GTP and 5 mM MgCl_2_ were added to the high speed supernatant (Input-sample) and protein concentration was determined using Bradford reagent (Biorad). The supernatants were warmed to 37°C for 5 minutes to allow microtubule polymerisation, and then taxol was added to a final concentration of 10 µM. Extracts were left at 37°C for further 10 minutes to stabilize microtubules, then transferred to RT for 30 min to allow binding of proteins to microtubules. Afterwards supernatants were centrifuged at 90.000 rpm (TLA100, Beckman) at 25°C for 5 min. Equal fractions of supernatant (SN) and pellet (P) were resuspended in protein sample buffer, boiled and subjected to SDS-PAGE.

### TIRF assays

Porcine brain tubulin was purified and rhodamine labelled (TAMRA, Invitrogen) as previously described [28]. 2 µM 30% rhodamine-labelled tubulin was polymerized in BRB80 (80 mM PIPES pH 6.9, 1 mM MgCl_2_, 1 mM EGTA) supplemented with 1 mM MgCL_2_, 2 mM GMPCPP overnight at 37 °C. After pelleting in an Airfuge (20 psi for 5 min; Beckman-Coulter) microtubules where resuspended in BRB80. Microscopy chambers were constructed as described [18], functionalized with anti-beta-tubulin antibody (SAP4G5) and passivated with 1% Pluronic F127 (Sigma) in BRB80. Microtubules were flowed into the chambers and allowed to bind for 5 min. Purified recombinant Kif18A(aa 1-898)-GFP or Kif18A(aa1-777)-GFP was diluted 2,000 to 20,000 times in motility buffer (BRB80, 110 KCl, 1 mM ATP, 1 mM DTT, 0.01% TWEEN-20, 80 µg/ml casein, anti-fade consisting of 20 mM glucose, 20 µg/ml glucose oxidase, 8 µg/ml catalase) and imaged at 28°C with a Zeiss Axiovert 200M TIRF microscope (equipped with Zeiss alpha-plan-Apochromat, 100 x, 1.46 oil; 488 nm Laser IonlaserTechnology, Andor iXon+ EMCCD camera operated with Metamorph). 600 images where taken with 150 ms exposure time at a rate of one image per second. Under these conditions there is little evidence of photobleaching, judged by the fluorescence lifetime of stationary motors. For observing 2-step bleaching, the ATP concentration in the motility solution was reduced to 15 µM to reduce the speed of the motor proteins and therefore increase their time on the microtubule before dissociating. TIRF imaging was done in streaming mode with an exposure time of 100 ms for 1200 frames. Only motors, which landed and dissociated within the kymograph were taken into account to reduce the effect of bleaching before recording. Diffusion was measured by recording Kif18A(aa 776-898)-GFP under single molecule conditions in Kif18A motility buffer. TIRF imaging was done in streaming mode with an exposure time of 100 ms and for 500 frames. Only for this imaging additional magnification of 2.5 was used. GFP fluorescence of purified recombinant Kif18A (aa 776-898)-GFP and Kif18A (R898)-GFP was determined with a Nanodrop and adjusted to similar fluorescence intensity. These samples were diluted 5 times in BRB80 and flowed into prepared imaging channels holding rhodamine-labelled microtubules. The presence of proteins in the sample solutions was confirmed on SDS gels.

## Supporting Information

Figure S1 **Kif18A^1-367^, Kif18A^1-467^ and Kif18A^1-526^ fail to properly localize to kt-MTs plus-ends. (A)** Localization of transiently GFP-Kif18A^1-367^ during metaphase in HeLa-cells treated with GL2 control siRNA determined by immunofluorescence. HeLa-cells were stained with CREST antisera (red), anti α-tubulin (blue), anti-pericentrin. Kif18A was visualized by GFP-tag. The scale bar is 15 µm. Images are z-projections of deconvolved 3D stacks. The merge image represents GFP, CREST and tubulin. **(B)** Lysates (input) were prepared from 293T cells transfected with empty GFP vector or the indicated Kif18A variants followed by immunoprecipitation of the fusion proteins with GFP antibodies. Western blots were probed with GFP and myc antibodies. **(C)** SDS-PAGE showing Kif18A^776-898^–GFP (42 kDa) and Kif18A^898^–GFP (28.3 kDa) as purified from bacteria. The 3C-Protease band (45 kDa) falls on top of the Kif18A^776-898^-GFP band. **(D)** SDS-PAGE showing His- Kif18A^1-777^-GFP (123 kDa) and His- Kif18A^FL^-GFP (137 kDa) as purified from insect cells. At∼75 kDa appears a typical insect-cell protein purification background band. **(E)** Kymographs of His-Kif18A^FL^-GFP molecules and His- Kif18A^1-777^-GFP recorded with TIRF microscopy in streaming mode under low ATP conditions. Arrows mark traces with bleaching events. Quantification states fractions observed among the traces that started and ended within one kymograph. **(F)** and **(G)** Fluorescence signal along a trace of moving His- Kif18A^1-777^-GFP and His- Kif18A^FL^-GFP molecules showing 2-step bleaching in a low ATP assay. Horizontal scale bars indicate 5 s, vertical scale bars indicate 1 µm.
(TIF)

Movie S1 Original movie sequence showing HeLa-cells stably expressing H2B-mRFP transfected with short interfering RNA-oligonucleotides (siRNAs) targeting luciferase (GL2) and transiently overexpressing GFP. Movies s1and s2 show H2B-mRFP and GFP, respectively. Selected frames of this movie are shown in Figure 3A.
(AVI)

Movie S2 Original movie sequence showing HeLa-cells stably expressing H2B-mRFP transfected with short interfering RNA-oligonucleotides (siRNAs) targeting luciferase (GL2) and transiently overexpressing GFP. Movies s1and s2 show H2B-mRFP and GFP, respectively. Selected frames of this movie are shown in Figure 3A.
(AVI)

Movie S3 Original movie sequence showing HeLa-cells stably expressing H2B-mRFP transfected with short interfering RNA-oligonucleotides (siRNAs) targeting Kif18A and transiently overexpressing GFP. Movies s3 and s4 show H2B-mRFP and GFP, respectively. Selected frames of this movie are shown in Figure 3B.
(AVI)

Movie S4 Original movie sequence showing HeLa-cells stably expressing H2B-mRFP transfected with short interfering RNA-oligonucleotides (siRNAs) targeting Kif18A and transiently overexpressing GFP. Movies s3 and s4 show H2B-mRFP and GFP, respectively. Selected frames of this movie are shown in Figure 3B.
(AVI)

Movie S5 Original movie sequence showing HeLa-cells stably expressing H2B-mRFP transfected with short interfering RNA-oligonucleotides (siRNAs) targeting Kif18A and transiently overexpressing GFP-Kif18A^FL^. Movies s5 and s6 show H2B-mRFP and GFP-Kif18A^FL^, respectively. Selected frames of this movie are shown in Figure 3C.
(AVI)

Movie S6 Original movie sequence showing HeLa-cells stably expressing H2B-mRFP transfected with short interfering RNA-oligonucleotides (siRNAs) targeting Kif18A and transiently overexpressing GFP-Kif18A^FL^. Movies s5 and s6 show H2B-mRFP and GFP-Kif18A^FL^, respectively. Selected frames of this movie are shown in Figure 3C.
(AVI)

Movie S7 Original movie sequence showing HeLa-cells stably expressing H2B-mRFP transfected with short interfering RNA-oligonucleotides (siRNAs) targeting Kif18A and transiently overexpressing GFP-Kif18A^1-777^. Movies s7 and s8 show H2B-mRFP and GFP-Kif18A^1-777^, respectively. Selected frames of this movie are shown in Figure 3D.
(AVI)

Movie S8 Original movie sequence showing HeLa-cells stably expressing H2B-mRFP transfected with short interfering RNA-oligonucleotides (siRNAs) targeting Kif18A and transiently overexpressing GFP-Kif18A^1-777^. Movies s7 and s8 show H2B-mRFP and GFP-Kif18A^1-777^, respectively. Selected frames of this movie are shown in Figure 3D.
(AVI)

Movie S9 Original movie sequence showing HeLa-cells stably expressing H2B-mRFP transfected with short interfering RNA-oligonucleotides (siRNAs) targeting Kif18A and transiently overexpressing GFP-Kif18A^778-898^. Movies s9 and s10 show H2B-mRFP and GFP-Kif18A^778-898^, respectively. Selected frames of this movie are shown in Figure 3E.
(AVI)

Movie S10 Original movie sequence showing HeLa-cells stably expressing H2B-mRFP transfected with short interfering RNA-oligonucleotides (siRNAs) targeting Kif18A and transiently overexpressing GFP-Kif18A^778-898^. Movies s9 and s10 show H2B-mRFP and GFP-Kif18A^778-898^, respectively. Selected frames of this movie are shown in Figure 3E.
(AVI)

Movie S11 Subnanomolar His-Kif18A^FL^-GFP moves uni-directionally along microtubules in vitro and is highly processive. TIRF microscopy pictures were taken at 1 frame per second. Video playback is 20x real-time.
(AVI)

Movie S12 Subnanomolar His-Kif18A^1-777^-GFP moves uni-directionally along microtubules in vitro. TIRF microscopy pictures were taken at 1 frame per second. Video playback is 20x real-time.
(AVI)
