# Supplementary figures and images for: A Non-Motor Microtubule Binding Site Is Essential for the High Processivity and Mitotic Function of Kinesin-8 Kif18A
Source: PLoS One. 2011 Nov 10;6(11):e27471. doi: 10.1371/journal.pone.0027471 (PMC3213134; doi:10.1371/journal.pone.0027471)

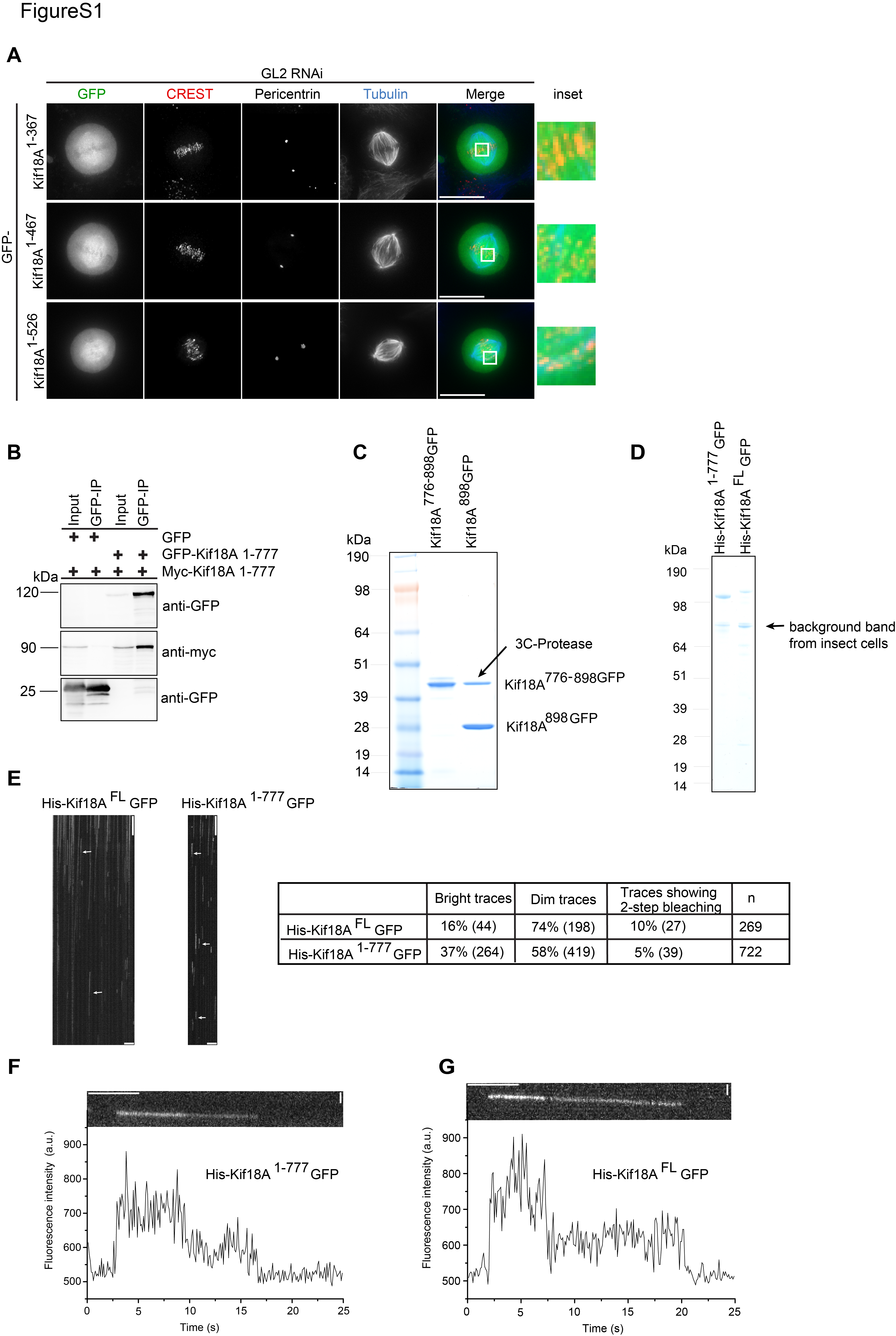

Supplement: Figure S1 — Kif18A1-367, Kif18A1-467 and Kif18A1-526 fail to properly localize to kt-MTs plus-ends. (A) Localization of transiently GFP-Kif18A1-367 during metaphase in HeLa-cells treated with GL2 control siRNA determined by immunofluorescence. HeLa-cells were stained with CREST antisera (red), anti α-tubulin (blue), anti-pericentrin. Kif18A was visualized by GFP-tag. The scale bar is 15 µm. Images are z-projections of deconvolved 3D stacks. The merge image represents GFP, CREST and tubulin. (B) Lysates (input) were prepared from 293T cells transfected with empty GFP vector or the indicated Kif18A variants followed by immunoprecipitation of the fusion proteins with GFP antibodies. Western blots were probed with GFP and myc antibodies. (C) SDS-PAGE showing Kif18A776-898–GFP (42 kDa) and Kif18A898–GFP (28.3 kDa) as purified from bacteria. The 3C-Protease band (45 kDa) falls on top of the Kif18A776-898-GFP band. (D) SDS-PAGE showing His- Kif18A1-777-GFP (123 kDa) and His- Kif18AFL-GFP (137 kDa) as purified from insect cells. At∼75 kDa appears a typical insect-cell protein purification background band. (E) Kymographs of His-Kif18AFL-GFP molecules and His- Kif18A1-777-GFP recorded with TIRF microscopy in streaming mode under low ATP conditions. Arrows mark traces with bleaching events. Quantification states fractions observed among the traces that started and ended within one kymograph. (F) and (G) Fluorescence signal along a trace of moving His- Kif18A1-777-GFP and His- Kif18AFL-GFP molecules showing 2-step bleaching in a low ATP assay. Horizontal scale bars indicate 5 s, vertical scale bars indicate 1 µm. (TIF) [file pone.0027471.s001.tif]
